# Congenital Corneal Anesthesia and Neurotrophic Keratitis: Diagnosis and Management

**DOI:** 10.1155/2015/805876

**Published:** 2015-09-16

**Authors:** Flavio Mantelli, Chiara Nardella, Eloisa Tiberi, Marta Sacchetti, Alice Bruscolini, Alessandro Lambiase

**Affiliations:** ^1^Department of Biology, College of Science and Technology, Temple University, 1900 N. 12 Street, Philadelphia, PA 19122, USA; ^2^Department of Sense Organs, Sapienza University, Viale del Policlinico 155, 00186 Rome, Italy; ^3^Neonatology Unit, Università Cattolica del Sacro Cuore Roma-Gemelli, Largo Agostino Gemelli 8, 00168 Rome, Italy; ^4^Cornea and Ocular Surface Unit, Ospedale San Raffaele IRCCS, Via Olgettina 60, 20132 Milan, Italy

## Abstract

Neurotrophic keratitis (NK) is a rare degenerative disease of the cornea caused by an impairment of corneal sensory innervation, characterized by decreased or absent corneal sensitivity resulting in epithelial keratopathy, ulceration, and perforation. The aetiopathogenesis of corneal sensory innervation impairment in children recognizes the same range of causes as adults, although they are much less frequent in the pediatric population. Some extremely rare congenital diseases could be considered in the aetiopathogenesis of NK in children. Congenital corneal anesthesia is an extremely rare condition that carries considerable diagnostic and therapeutic problems. Typically the onset is up to 3 years of age and the cornea may be affected in isolation or the sensory deficit may exist as a component of a congenital syndrome, or it may be associated with systemic somatic anomalies. Accurate diagnosis and recognition of risk factors is important for lessening long-term sequelae of this condition. Treatment should include frequent topical lubrication and bandage corneal or scleral contact lenses. Surgery may be needed in refractory cases. The purpose of this review is to summarize and update data available on congenital causes and treatment of corneal hypo/anesthesia and, in turn, on congenital NK.

## 1. Introduction

The cornea is the tissue with the richest innervation in the human body. Marfurt et al. [1] showed that around 70 nerve bundles enter the cornea at the corneoscleral limbus and then give rise through repetitive branching to a moderately dense midstromal plexus and a dense subepithelial plexus. It is well known that the trigeminal nerve is responsible for providing sensitivity to the cornea but also for providing a trophic support through the release of neurotrophic factors that play a fundamental role in maintaining its anatomical integrity, transparency, and function.

The ophthalmic branch of the trigeminal nerve has 2 reflex arcs: a motor arc that regulates eyelid movements (i.e., blinking) and an autonomic arc that regulates the secretion of goblet cells and lacrimal and meibomian glands. The integration of these two reflex arcs is responsible for the production, maintenance, and stability of the preocular tear film, which is also responsible for providing a trophic support to the cornea. Therefore, the impairment of corneal sensory innervation is overall devastating as it triggers a detrimental loop in which a reduction in trophic support to the tissue is accompanied by an aberrant reduction in the lacrimation reflex and in blinking, with a consequent damage to epithelial cells, which are also burdened by a parallel deficiency in spontaneous epithelial repair [2–5].

Patients suffering decrease or absence of corneal sensitivity develop a clinical condition called neurotrophic keratitis (NK), also known as neurotrophic keratopathy or neuroparalytic keratitis: regardless of the underlying cause, NK is a rare degenerative disease of the cornea caused by an impairment of corneal sensory innervation, characterized by decreased or absent corneal sensitivity (hypo/anaesthesia), resulting in spontaneous epithelial breakdown and reduced corneal healing [6].

NK can be caused by systemic, ocular, congenital, or iatrogenic diseases that lead to a damage to the fifth cranial nerve.

## 2. Aetiopathogenesis

Although a wide range of ocular and systemic diseases may cause neurotrophic keratitis, one common insult is always present: a lesion of the fifth (trigeminal) cranial nerve or its ophthalmic branch [6].

The most common causes of neurotrophic keratitis are viral infections (herpes simplex and herpes zoster keratoconjunctivitis) [7, 8], followed by surgical interventions to the trigeminal nerve or for acoustic neuroma [9]. In fact, neurosurgical procedures can cause an insult and consequent damage to the trigeminal nucleus, root, or ganglion, or also directly to the ophthalmic branch of the nerve [10, 11]. Toxicity from chronic use of topical ocular medications may also cause nerve damage and result in corneal hypo/anaesthesia [12, 13]. Neurotrophic keratitis has also been associated with systemic diseases such as diabetes mellitus [14, 15]. A complete list of all known causes of NK is provided in Table 1.

Generally speaking, the aetiopathogenesis of corneal sensory innervation impairment in children recognizes the same range of causes as adults, although they are much less frequent in the pediatric population. In fact, diseases such as uncontrolled diabetes and advanced multiple sclerosis and leprosy are very unrealistic in children, and even herpes simplex infection, which may occur in children, needs a long history of recurrences before inducing damage to the corneal nerves. In addition, it must be considered that corneal and refractive surgery, as well as abuse of local anesthetics [23, 24], chronic topical glaucoma therapy [25, 26], and chronic contact lens wear [27], which are amongst the most common iatrogenic causes of NK in adults, play a very limited role in childhood.

On the other hand, some extremely rare congenital diseases could be considered in the aetiopathogenesis of NK in children. Among them, the rare congenital corneal anesthesia could be a cause of corneal ulceration and scarring in children. Typical onset is up to 3 years of age, more commonly between the age of 8 and 12 months. It may occur as an isolated disease or associated with systemic diseases such as familial dysautonomia (Riley-Day syndrome), Goldenhar syndrome, Möbius syndrome, and congenital insensitivity to pain. Typically, the congenital corneal anesthesia occurs bilaterally; one-sided cases, however, are also described. The cornea may be affected in isolation or as part of a sensory deficit of the 1st and 2nd (and possibly 3rd) branch of the trigeminal nerve [5, 6].

## 3. Congenital Causes of the Disease

Only scattered NK cases have been reported, highlighting the rarity of NK development in congenital syndromes that are rare* per se*. Those include the following congenital diseases, all considered rare diseases.

### 3.1. Familial Dysautonomia (Riley-Day Syndrome)

The Riley-Day syndrome belongs to the hereditary sensory and autonomic neuropathies (HSAN) and according to the numerical classification of four distinct forms of HSAN that was proposed by Axelrod and Gold-von Simson it is also known as HSAN III [28]. It is an inherited autosomal recessive disorder that affects the development and function of nerves throughout the body, with a prevalence of less than 1 on 1,000,000. This condition is almost exclusive to individuals of Eastern European Jewish ancestry (Ashkenazi Jews), and the incidence is 1 in 3,600 live births [28]. The carrier frequency in this population is about 1/30. If both parents are carriers, there is a 25% chance with each pregnancy to give birth to an affected child. Worldwide, there have been approximately 600 diagnoses recorded since the discovery of the disease, with approximately 350 of the affected individuals still living [29].

Corneal ulcerations have been reported in up to 50% of patients [30]. However, it must be noted that this disease is characterized by congenital alacrima, resulting in severe dry eye rather than NK, and it has never been reported that the corneal lesions in affected patients are NK ulcers: there is only one Riley-Day syndrome patient reported in the scientific literature with a corneal epithelial defect caused by “loss of corneal sensation and hypolacrimation” [31]. Therefore, it cannot be determined whether this lesion was a NK PED or a dry eye epithelial damage. Even if we assume that this was a case of NK, the impact of Riley-Day syndrome on the overall NK prevalence can safely be considered negligible.

### 3.2. Goldenhar-Gorlin Syndrome (Oculo-Auriculo-Vertebral Spectrum)

This syndrome was first described by Canton in 1861 and Von Arlt in 1881, but only in 1952 Goldenhar-Gorlin provided a comprehensive description. It is a rare congenital defect (prevalence 1–9 in 100,000) which manifests with a number of craniofacial abnormalities that affect the structures embryologically derived from the first and second branchial arches and usually involve the face (hemifacial microsomia) and ears (microtia). In 50% of cases other anomalies to heart and eyes (epibulbar dermoid) can be found. Most cases are sporadic with 1-2% cases transmitted as autosomal dominant (in this case the most likely affected region is 14q32 and the male to female ratio is 2 : 1). It affects between 1/5000 and 1/25000 live births [32]. Overall, the ophthalmic anomalies involve approximately 66% of cases, with epibulbar dermoids (28–35%), blepharoptosis (10%), superior palpebral coloboma (20%), and microphthalmia (18%). Corneal manifestations are thought to be related to both decreased tear production and neuroparalytic keratitis, possibly due to aplasia or hypoplasia of the trigeminal nuclei, as can be retrieved from case reports involving a few children or young adults [33–35]. In addition, an isolated involvement of the facial nerve can be observed in approximately 20% of cases.

The series published by Mansour et al. [36] does not mention NK as one of the ocular manifestations of this syndrome in 57 consecutive patients. In fact, only one case of corneal anesthesia associated with epithelial punctate keratopathy with this disease has ever been reported [37]. The typical asymmetric facial presentation of this syndrome suggests that this could be a case of exposure keratopathy; however, even if we assume that this was a case of NK, the impact of Goldenhar-Gorlin syndrome on the overall NK prevalence can safely be considered negligible.

### 3.3. Möbius Syndrome

This extremely rare disease was first described by the ophthalmologist P.J. Möbius in 1888, who described a few patients with the concomitant presence of a nonprogressive congenital paralysis of the facial and abducens nerves. The actual prevalence is unknown; however, only approximately 300 cases have been reported in the literature to date with no differences in gender [38]. Most cases of Möbius syndrome are isolated sporadic cases with no notable family history. It is estimated that there are, on average, 2 to 20 cases per million births. In a study conducted on the Dutch population in 1996, an incidence of 1/189000 live births was found [39]. It must be noted that in this syndrome it is not the trigeminal nerve, but the facial nerve, that is involved. Specifically, the Möbius syndrome is caused by an abnormal development of the 7th cranial nerve (facial) in all patients and of the 6th cranial nerve (abducens) in 75% of the cases. Only in a minority of cases other cranial nerves, including the trigeminal nerve, can be involved. Therefore, it is more likely that a corneal lesion, in the context of this syndrome, is caused by exposure keratopathy (by facial paresis) and not by NK. While it is theoretically possible that a subset of patients affected by Möbius syndrome with trigeminal involvement have corneal hypesthesia, NK cases have not yet been described and, therefore, there is no impact of this syndrome on the overall NK prevalence. On the other hand, other ophthalmic anomalies have been described in this syndrome, including congenital ptosis, epicanthus, and hypertelorism.

### 3.4. Familial Corneal Hypesthesia (Familial Trigeminal Anesthesia)

The prevalence of this condition is unknown, and only scattered case reports have been published in scientific literature. However, these reports do not differentiate between isolated trigeminal anesthesia and corneal hypesthesia associated with the other syndromes listed above [40]. Therefore, the impact of familial trigeminal anesthesia on the overall NK prevalence can safely be considered negligible.

### 3.5. Congenital Insensitivity to Pain with Anhidrosis (CIPA)

The exact prevalence of this rare condition is unknown. According to scientific literature only 52 cases of CIPA have ever been reported worldwide, of which only 14 had corneal involvement [41]. Considering the extreme rarity of this condition, even if we assume that the cornea was involved in all cases during the patients' life, the impact of CIPA on the overall NK prevalence can safely be considered negligible.

However, the early diagnosis and prompt treatment of this rare condition are mandatory to prevent corneal complications such as scarring and perforation.

Regardless of the cause behind the damage to corneal sensory innervation, once NK develops, the corneal epithelium is the first target of the disease, which begins with dystrophic changes of the epithelial cells and, later, with frank epithelial defects that have a poor tendency to spontaneous healing. Epithelial damage of increasing severity is observed according to the stage of the disease. Progression of the disease can lead to corneal ulceration, infection, melting, perforation, and, ultimately, loss of sight [6].

## 4. Clinical Staging of the Disease

According to the Mackie classification, it is possible to classify neurotrophic keratitis into three stages (1 to 3 in order of increasing severity) [6].

- *Stage 1* is characterized by punctate keratopathy and/or corneal epithelial hyperplasia and irregularity, which may be associated with superficial neovascularization and stromal scarring. In addition dry eye signs may be observed, including vital dye (such as rose bengal) staining of the inferior palpebral conjunctiva and decreased tear film break-up time.
- *Stage 2* is characterized by a persistent corneal epithelial defect (PED), typically oval or circular in shape, with smooth and rolled edges. An area of poorly adherent opaque and oedematous epithelium is typically found rolled-up around the margin of the epithelial defect. As this loose epithelium can easily detach spontaneously, a rapid enlargement of the defect is often observed. Oedema of the corneal stroma may also be present, and it is not uncommon to observe also an inflammatory reaction in the anterior chamber.
- *Stage 3* often ensues if stages 1 and 2 are not treated appropriately. In this stage the corneal stroma is involved and a corneal ulcer is observed. Corneal ulceration tends to progress to perforation and/or stromal melting if not promptly and properly treated. Corneal melting and perforation can be also iatrogenically caused by inappropriate use of topical steroids or by secondary infections of the nonhealing ulcer.

## 5. Clinical Diagnosis of the Disease

The medical history and clinical findings are crucial for making a proper diagnosis. Anamnesis often highlights systemic diseases, previous neural surgeries, recurrent corneal herpetic infections, trauma, and topical drug abuse. Other characteristic observations include a disproportion between clinical signs as observed at the slit lamp and symptoms as referred by the patient, who is often completely asymptomatic. In fact, corneal sensitivity is a key information to orientate the diagnosis. Obviously, considering all this, it is clear that making a diagnosis of congenital corneal anesthesia and NK is extremely challenging in young children, unless the diagnosis of a well-known syndrome causing corneal anesthesia is confirmed. In fact, in adult, collaborative patients, a qualitative assessment of corneal sensitivity reduction can be easily measured using a piece of twisted cotton, but a quantitative evaluation using a Cochet-Bonnet or no-contact gas esthesiometer is preferred and should be mandatory. Cochet-Bonnet esthesiometry is the most widely used method and it is performed by touching the central and peripheral cornea with a nylon thread that can be elongated up to 60 mm (the longer the thread, the lighter the touch on the cornea) and by recording the patient's response. All the different quadrants of the cornea have to be tested separately because in some cases, including herpes simplex and zoster keratitis, the impaired corneal sensitivity may be only sectorial [6]. This test is difficult to perform, if possible at all, in young children, who are not collaborative and are not able to tell to the ophthalmologist at which length of the Cochet-Bonnet thread they feel the corneal sensation upon touching. The use of in vivo corneal confocal microscopy to visualize the altered subepithelial nerve plexus is also not feasible in young children.

Slit lamp examination can be of great help, especially in children, to evidence the characteristics corneal lesions according to disease severity. Iris atrophy may be a sign of previous herpes infection or previous intraocular inflammation, which sometimes is observed in stage 2 NK patients [42]. There may be optic nerve pallor or swelling due to an intracranial tumor that causes trigeminal compression and impairment; therefore, a complete visit including dilated fundus oculi examination must always be carried out; in the case of visualization of optic nerve head changes it is always important to exclude glaucoma by intraocular pressure testing and visual field examination [43, 44].

Additionally, a clinical evaluation of the different cranial nerves' function can help localize the cause of the decreased corneal sensation. For instance, an isolated dysfunction of VII and VIII cranial nerves may indicate an acoustic neuroma or damage from surgical resection of the lesion, while the paresis of cranial nerves III, IV, and VI may indicate an aneurysm or cavernous sinus pathology that can also affect the trigeminal nerve.

Lastly, the eyelids also need to be carefully examined for both diagnostic and prognostic reasons. In fact, eyelids that do not close properly not only indicate a cranial nerve VII palsy and help in the disease diagnosis but also can worsen the clinical picture and accelerate progression towards stage 3 disease for epithelial exposure by lagophthalmos [6].

Corneal anaesthesia is the hallmark of NK and, therefore, the presence of ocular symptoms such as burning, foreign body sensation, photophobia, and dry eye easily orientates the diagnosis towards other ocular surface diseases. Infective, toxic, or immune corneal ulcers also differ from NK as they are typically accompanied by ocular inflammation and stromal infiltrates. To exclude the presence of an infective ulcer, a microbiologic examination needs to be performed (in the presence of a nonhealing corneal lesion, not only if infiltrates are present, microbiologic exams should always be performed). In order to exclude toxic corneal ulcers, all topical treatments must be discontinued. In order to exclude immune corneal ulcers, a thorough systemic evaluation for immune disorders should also be carried out.

Since superficial corneal vascularisation and epithelial defects can also be seen in limbal stem cell deficiency an additional examination that can be performed in doubtful cases is impression cytology, a minimally invasive technique that allows the direct visualization and identification of the corneal or conjunctival epithelial phenotype by Periodic Acid Schiff staining or immunostaining for cytokeratins [6].

## 6. Prognosis of the Disease

The prognosis of neurotrophic keratitis depends upon a wide range of factors, including the specific cause behind corneal sensitivity impairment, the degree of corneal hypo/anesthesia, and the association with other ocular surface diseases such as dry eye, exposure keratitis, and limbal stem cell deficiency. The prognosis of congenital NK is usually poor as no medically effective therapy is currently available and due to the chronic and degenerative nature of the disease most patients are likely to end up developing disease complications or associated ocular surface alterations.

While the presence of associated ocular surface diseases may differently affect the prognosis, it is well known that usually the more severe is the corneal sensory impairment, the higher is the rapidity of disease progression towards corneal melting, perforation, and sight loss due to anatomical loss of the eye or permanent loss of corneal transparency [6].

Nevertheless, even in patients that do not have a complete corneal anesthesia or associated diseases such as secondary bacterial infection, and sometimes even despite early and appropriate therapy, neurotrophic keratitis may still progress to stage 3 (corneal ulcer) disease. When a neurotrophic corneal ulcer develops, it always requires prompt action in order to stop the stromal lysis and prevent perforation. Unfortunately, even when corneal perforation is avoided, once a corneal ulcer develops a permanent decrease in visual acuity from corneal scarring and astigmatism can still be the final outcome of the disease [6].

## 7. Prevention

Given the wide range of underlying pathologies observed in NK, no consistent approach to prevention is realistic. The most important preventive approach is the prompt identification of stage 1 patients who can be addressed with intense and continuous ocular lubrication with preservative free compounds. The prevention of disease progression with the use of therapeutic contact lenses is also a valid approach for small persistent epithelial defects.

## 8. Treatment

NK treatment largely depends on disease stage and severity. In fact, the therapeutic approach for stage 1 disease is mostly aimed at preventing epithelial breakdown, while treatment for stages 2 and 3 is mostly aimed at facilitating healing of the corneal lesion in order to prevent irreparable consequences on visual function. More specifically, in the presence of a persistent epithelial defect, treatment aims at preventing stromal involvement with corneal ulcer formation, while more advanced cases, with the presence of a corneal ulcer and stromal melting, require immediate attention to stop the stromal lysis and prevent corneal perforation. This is often obtained by surgery.

Although pharmacological treatments for NK are being intensively studied, with currently ongoing phase 2 clinical trials evaluating growth factors and neuropeptides in both Europe and USA [31, 45], none are yet available. However, the use of preservative-free artificial tears may help improve the corneal surface at all stages of disease, and continuous lubrication with preservative-free compounds needs to be recommended, especially in children, not only to stage 1 patients. The use of preservative free topical antibiotic eye drops is often encouraged and recommended in children to prevent superinfections in eyes with NK at stages 2 and 3; however, it must also be kept in mind that NK lesions are noninfective and, therefore, the use of topical antibiotics is only preventive. When ocular inflammation is present, especially in the case of hypopyon visualization in the anterior chamber, topical steroids have been proposed for NK and may be highly necessary; however, their use is very controversial and must be extremely cautious since steroids may increase the risk of corneal melting and perforation by inhibiting stromal healing. Topical nonsteroidal anti-inflammatory drugs may also inhibit the healing process and should be avoided since their added benefit to control anterior chamber inflammation would be minimal. Regardless of the concomitant use of topical steroids, in the event of stromal melting, the use of topical collagenase inhibitors such as N-acetylcysteine [46] and systemic administration of tetracycline or medroxyprogesterone has been suggested and is usually considered a valid therapeutic option [47]. Nonpharmacological treatments for NK include therapeutic corneal or scleral contact lenses in the event of PED to promote corneal epithelial healing and postpone severe corneal complications [48]. Prolonged therapeutic contact lens use may increase the risk of secondary infections and the concomitant use of topical antibiotics is mandatory [49].

Surgical treatments should be avoided, especially in children, as they are all burdened by a reduction in visual function. Therefore, surgery is usually reserved for refractory cases or for cases that show rapid disease progression towards stromal thinning despite all the best therapeutic efforts. Partial or total tarsorrhaphy is the most simple and widespread procedure used to promote corneal healing in the presence of a neurotrophic PED. Surgical tarsorrhaphy may be performed easily and the tarsorrhaphy opening may be enlarged a few weeks after corneal healing; however, opening a tarsorrhaphy prematurely often results in an early disease recurrence. Alternatively to the tarsorrhaphy, the use of botulinum A toxin injection of the eyelid elevator muscle has been proposed to cover the PED and promote healing [50].

Amniotic membrane transplantation (AMT) is a surgical technique for ocular surface reconstruction that carries relatively good results and can be considered in the management of refractory neurotrophic corneal ulcers. AMT is relatively easy to perform and is effective in promoting corneal epithelial healing, reducing vascularisation, and reducing ocular surface inflammation. A multilayer AMT has also been proposed for treating deeper neurotrophic corneal ulcers [51].

The conjunctival flap is a more invasive procedure that should be restricted only to very severe cases with impending perforations. In fact, although this surgical procedure is very effective as it is able to restore ocular surface integrity and provide metabolic and mechanical support for corneal healing, it also severely compromises visual function and the esthetic outcome is also very poor: in this procedure, the deep corneal ulcer (or corneal perforation) is covered by a pedunculated conjunctival flap secured in place by fine sutures that provides vascular support to the nonhealing cosmetic cornea. The main goal of this procedure is to preserve the anatomical integrity of the eye, but it does obviously severely impact visual function since the flap vascularizes [52, 53].

In the presence of a small perforation (less than 3 mm) a less invasive approach can be used, by the application of cyanoacrylate glue on the lesion, followed by the application of a soft bandage contact lens or AMT. Larger defects require a conjunctival flap or lamellar keratoplasty; however, it must be kept in mind that the success rate of corneal transplants in NK patients is very low due to the lack of trophic support, with consequent poor wound healing and risk of PED/ulcer recurrence [54].

In children with congenital corneal anesthesia or other forms of disease that may cause NK, a prompt diagnosis and a life-long monitoring and therapy are important to prevent amblyopia and permanent visual damage. The main goal of therapy is to prevent the formation of epithelial defects. An intensive lubrication is essential. The wearing of safety glasses to avoid self-inflicted injuries is also generally recommended in young children.

If persistent epithelial defects or frank corneal ulcers develop, a fast and aggressive therapy is necessary in order to avoid a corneal perforation. In persistent epithelial defects, soft highly hydrophilic contact lenses can be used together with prophylactic application of antibiotic unpreserved eye drops.

When ulcers develop, amniotic membrane transplantation may be considered in children. Tarsorrhaphy and a protective ptosis by Botulinum toxin injection in the levator palpebrae can be considered in more severe cases [55, 56], but the amblyogenic potential of all these treatments must always be considered in young children.

## 9. Conclusions

The diagnosis of congenital corneal anesthesia is a real challenge for ophthalmologists because the typical diagnostic tests, such as Cochet-Bonnet corneal esthesiometry, are often not feasible in young children. Therefore, it is mandatory for the ophthalmologists to be aware of the rare systemic disorders that can cause congenital corneal anesthesia and NK, including the Riley-Day, Möbius, and Goldenhar syndromes. Once corneal anesthesia is suspected or a corneal lesion is observed in the affected children, the clinical management is also a real challenge. In fact, although a number of promising clinical trials are ongoing to test therapeutic interventions for NK, there is currently no medication available to restore corneal sensitivity and cure the disease. All current medical treatments, from intense ocular lubrication to therapeutic contact lens and to autologous serum eye drops, are useful to prevent or slow down progression in patients diagnosed with stage 1 or early stage 2 disease. However, once a large epithelial defect or a frank neurotrophic corneal ulcer is present, surgery is often needed to prevent further disease progression and avoid corneal perforation. Unfortunately, surgical interventions such as tarsorrhaphy, amniotic membrane transplantation, or conjunctival flap inevitably cause a decrease in visual function, which can result in amblyopia when performed in young children.

## Figures and Tables

**Table 1:** Aetiopathogenesis of neurotrophic keratitis.

Infections	Herpes simplex Herpes zoster Leprosy [16]

Corneal pathologies	Dystrophies (i) Lattice (ii) Granular

Iatrogenic injury	Contact lens wear Surgeries Laser-assisted in situ keratomileusis [17] Corneal incision [18] Lamellar and penetrating keratoplasty

Topical medications	Anaesthetics Timolol Betaxolol Trifluridine Sulfacetamide Diclofenac sodium [19]

Toxic substances	Chemical burns Exposure to oleoresin capsicum pepper spray [20] Exposure to hydrogen sulfide (H2S) [21]

Cranial nerve V palsy	Trigeminal neuralgia surgery Neoplasm Aneurysm Facial trauma [10] Congenital: (i) Riley-Day syndrome (ii) Möbius corneal hypoesthesia (iii) Goldenhar syndrome (iv) Familial corneal hypesthesia (v) (Familial trigeminal anesthesia) (vi) Congenital insensitivity to pain with anhidrosis

Systemic diseases	Diabetes Vitamin A deficiency Multiple sclerosis

Miscellaneous	Increasing age Adie's syndrome [22]
